# Neutrophil activation in patients with anti-neutrophil cytoplasmic autoantibody-associated vasculitis and large-vessel vasculitis

**DOI:** 10.1186/s13075-022-02849-z

**Published:** 2022-06-29

**Authors:** Despina Michailidou, Bhargavi Duvvuri, Runa Kuley, David Cuthbertson, Peter C. Grayson, Nader A. Khalidi, Curry L. Koening, Carol A. Langford, Carol A. McAlear, Larry W. Moreland, Christian Pagnoux, Philip Seo, Ulrich Specks, Antoine G. Sreih, Kenneth J. Warrington, Tomas Mustelin, Paul A. Monach, Peter A. Merkel, Christian Lood

**Affiliations:** 1 Division of Rheumatology, Department of Medicine, University of Washington, 750 Republican Street, Seattle, WA 98109 USA; 2 Health Informatics Institute, University of South Florida, South Florida, Tampa, FL USA; 3 Systemic Autoimmunity Branch, National Institutes of Arthritis and Musculoskeletal and Skin Diseases, Bethesda, MD USA; 4 Division of Rheumatology, Mc Master University, Hamilton, Ontario Canada; 5 Division of Rheumatology, University of Utah, Salt Lake City, UT USA; 6 Division of Rheumatology, Cleveland Clinic, Cleveland, OH USA; 7 Division of Rheumatology, University of Pennsylvania, Philadelphia, PA USA; 8 Division of Rheumatology and Clinical Immunology, University of Colorado, Denver, CO USA; 9 Division of Rheumatology, Mount Sinai Hospital, Toronto, Canada; 10 Division of Rheumatology, Johns Hopkins University, Baltimore, MD USA; 11 Division of Pulmonary and Critical Care Medicine, Mayo Clinic, Rochester, MN USA; 12 Division of Rheumatology, Mayo Clinic, Rochester, MN USA; 13 Division of Rheumatology, Brigham and Women’s Hospital, Boston, MA USA

**Keywords:** Anti-neutrophil cytoplasmic antibody-associated vasculitis, Large-vessel vasculitis, Neutrophils, Mitochondria, Formyl peptide receptor 1

## Abstract

**Objective:**

To assess markers of neutrophil activation such as calprotectin and N-formyl methionine (fMET) in anti-neutrophil cytoplasmic autoantibody-associated vasculitis (AAV) and large-vessel vasculitis (LVV).

**Methods:**

Levels of fMET, and calprotectin, were measured in the plasma of healthy controls (*n*=30) and patients with AAV (granulomatosis with polyangiitis (GPA, *n*=123), microscopic polyangiitis (MPA, *n*=61)), and LVV (Takayasu’s arteritis (TAK, *n*=58), giant cell arteritis (GCA, *n*=68)), at times of remission or flare. Disease activity was assessed by physician global assessment. In vitro neutrophil activation assays were performed in the presence or absence of formyl peptide receptor 1 (FPR1) inhibitor cyclosporine H.

**Results:**

Levels of calprotectin, and fMET were elevated in patients with vasculitis as compared to healthy individuals. Levels of fMET correlated with markers of systemic inflammation: C-reactive protein (*r*=0.82, *p*<0.0001), and erythrocyte sedimentation rate (*r*=0.235, *p*<0.0001). The neutrophil activation marker, calprotectin was not associated with disease activity. Circulating levels of fMET were associated with neutrophil activation (*p*<0.01) and were able to induce de novo neutrophil activation via FPR1-mediated signaling.

**Conclusion:**

Circulating fMET appears to propagate neutrophil activation in AAV and LVV. Inhibition of fMET-mediated FPR1 signaling could be a novel therapeutic intervention for systemic vasculitides.

## Introduction

Neutrophils are important mediators of the innate immune system and play a significant role in host defense against pathogenic microorganisms [1], via their recruitment at sites of tissue infection where they phagocytize pathogens, produce reactive oxygen species (ROS), and release neutrophil extracellular traps (NETs) [2]. NETs are webs of extruded chromatin, citrullinated histones, and granular components, including proteinase 3 (PR3), neutrophil elastase (NE), calprotectin (also known as S100A8/A9 or myeloid-related protein 8/14 (MRP8/14)), and myeloperoxidase (MPO) [3, 4]. NETs contribute to the pathogenesis of many autoimmune diseases [5–7], including anti-neutrophil cytoplasmic autoantibody (ANCA)-associated vasculitis (AAV) [8], a disease of small blood vessels characterized by the presence of circulating ANCA [9]. AAV includes three different subtypes, namely granulomatosis with polyangiitis (GPA), microscopic polyangiitis (MPA), and eosinophilic granulomatosis with polyangiitis (EGPA) [10].

In AAV, neutrophil activation by ANCA depends on priming by inflammatory cytokines like tumor necrosis factor-alpha (TNF-α), lipopolysaccharide (LPS), or complement factor 5a (C5a). These stimuli lead to externalization of the antigens MPO, and PR3 to the neutrophil cell surface where ANCA can bind to them. Upon binding, the Fc part of the autoantibody crosslinks FcγRs, resulting in profound neutrophil activation, NET formation, and inflammatory damage to the endothelium [11–13]. However, the role of neutrophil activation and NET formation in large-vessel vasculitis (LVV), which includes Takayasu’s arteritis (TAK) and giant cell arteritis (GCA), characterized by vascular inflammation and damage of the aorta and/or major branch arteries [14, 15], is still unknown.

NETs release mitochondrial components that signal through DNA sensing TLR9 as well as the cGAS-STING pathway [16]. Mitochondrial-derived N-formyl methionine peptides (fMET) are known potent neutrophil chemoattractants [17, 18]. fMET acts primarily through a highly expressed G-protein coupled receptor, formyl peptide receptor 1 (FPR1), inducing neutrophil chemotaxis, degranulation, and reactive oxygen species (ROS) generation [19–22]. Blockade of the neutrophil receptor FPR1 with the selective antagonist cyclosporine H (CsH) impairs neutrophil chemotaxis towards necrotic cells [23].

Although fMET plays a role in the pathogenesis of many inflammatory diseases such as inflammatory bowel diseases [24, 25], and rheumatoid arthritis (RA) contributing to neutrophil-mediated inflammation and disease progression [26], the role of fMET in the pathogenesis of vasculitides remains unclear.

In the current study, we first investigated levels of the neutrophil activation marker calprotectin, as well as levels of fMET, in a large cohort of patients with AAV and LVV. Secondly, we assessed whether levels of neutrophil activation were associated with disease activity and inflammatory markers. Finally, we investigated whether fMET/FPR1-mediated signaling can drive neutrophil activation in patients with vasculitis.

## Materials and methods

### Patient characteristics

Plasma samples from patients with GPA (123 patients in remission, 73 paired samples with flare), MPA (61 patients in remission, 11 paired samples with flare), TAK (58 patients in remission, 8 paired samples with flare), and GCA (68 patients in remission, 18 paired samples with flare) who had been enrolled in the Vasculitis Clinical Research Consortium (VCRC) were analyzed in this study. Demographic data, including disease subgroups, gender, age at diagnosis, and disease duration for the study populations in remission and active disease were also recorded (Tables 1 and 2, respectively). For the GPA and MPA cohorts, the prevalence of anti-MPO and anti-PR3 antibodies is also detailed.

**Table 1:** Demographic characteristics of study populations in remission

Diagnosis	GPA	MPA	TAK	GCA
Number of subjects	123	61	58	68
Gender (female, %)	64 (52%)	31 (51%)	53 (91%)	49 (72%)
Age at diagnosis, years (median, range)	47 (10–80)	59 (17–82)	30(12–58)	69 (54–90)
Disease duration (mean ± SD, years)	8.2 ± 7.5	4.8 ± 5.2	10.4 ± 7.2	3.4 ± 3.6
ESR (mean ± SD, mm/h)	12.8 ± 10.8	18.9 ± 15.3	15.2 ± 15.0	14.1 ± 13.1
CRP (mean ± SD, mg/dl)	6.7 ± 8.8	4.6 ± 5.4	6.9 ± 9.1	6.3 ± 7.6
Creatinine (mean ± SD, mg/dl)	1.11 ± 0.60	1.61 ± 0.96	0.82 ± 0.20	0.92 ± 0.20
Anti-MPO antibodies (%)	20 (16%)	54 (89%)	ND	ND
Anti-PR3 antibodies (%)	86 (70%)	8 (13%)	ND	ND
PGA (mean ± SD)	0	0	0	0

*GPA* granulomatosis with polyangiitis, *MPA* microscopic polyangiitis, *TAK* Takayasu’s arteritis, *GCA* giant cell arteritis, *SD* standard deviation, *ESR* erythrocyte sedimentation rate, *CRP* c-reactive protein, *PGA* physicians global assessment, *ND* no data

**Table 2:** Demographic characteristics of study populations in active disease

Diagnosis	GPA	MPA	TAK	GCA
Number of subjects	73	11	8	18
Gender (female, %)	38 (52%)	6 (55%)	8 (100%)	12 (67%)
Age at diagnosis, years (median, range)	47 (10–80)	67 (28–75)	28 (14–44)	69 (58–83)
Disease duration (mean ± SD, years)	6.8 ± 7.6	4.0 ± 5.2	6.9 ± 4.1	1.6 ± 1.8
ESR (mean ± SD, mm/h)	17.2 ± 13.9	31.1 ± 37.3	27.7 ± 22.3	16.2 ± 18.6
CRP (mean ± SD, mg/dl)	11.7 ± 22.7	17.2 ± 39.1	11.6 ± 13.3	11.7 ± 18.3
Creatinine (mean ± SD, mg/dl)	1.12 ± 0.77	1.98 ± 1.12	0.76 ± 0.13	0.92 ± 0.23
Anti-MPO antibodies (%)	6 (8%)	11 (100%)	ND	ND
Anti-PR3 antibodies (%)	55 (75%)	0 (0%)	ND	ND
PGA (mean ± SD)	3.30 ± 1.73	4.09 ± 1.92	3.88 ± 1.46	2.78 ± 1.22

*GPA* granulomatosis with polyangiitis, *MPA* microscopic polyangiitis; *TAK* Takayasu’s arteritis, *GCA* giant cell arteritis, *SD* standard deviation, *ESR* erythrocyte sedimentation rate, *CRP* c-reactive protein, *PGA* physicians global assessment, *ND* no data

Disease activity was assessed using physician global assessment (PGA) in all patients with vasculitis. PGA correlates well with the Birmingham Vasculitis Activity Score (BVAS) [27]. For all patients, laboratory data such as creatinine, and markers of systemic inflammation (CRP and ESR) were collected. Additionally, for patients with GCA, overlap with polymyalgia rheumatica (PMR) was recorded.

Plasma from healthy individuals (HC, *n*=30) were recruited through the University of Washington. The study was approved by the appropriate local institutional review boards at the University of Washington, Seattle, WA, (#3100), and informed consent was obtained from all participants in accordance with the Helsinki Declaration.

### ELISA assays

Plasma levels of human N-formyl methionine (My BioSource Inc., San Diego, CA, USA) and calprotectin (R&D Systems, Minneapolis MN, USA)) were measured by ELISA following the manufacturers’ instructions. Absorbance was measured by a plate reader at 450 nm (Synergy 2, BioTek).

### Neutrophil isolation

Heparinized blood from healthy individuals was layered on Polymorphprep (Axis-Shield, Dundee, UK) density gradient, according to the manufacturer’s instructions, or as described previously [28–30]. Red blood cells were lysed using RBC lysis buffer (BioLegend, San Diego, CA, USA). Neutrophils were re-suspended in serum-free RPMI-1640 medium (Life Technologies, Waltham, MA) for in vitro assays.

### fMET-mediated release of calprotectin from neutrophils

Neutrophils, 1–5 × 10^6^ in 200 μl, from healthy individuals were incubated with fMLP (10 nM) at 37°C for 60 min. As a negative control, neutrophils were incubated with RPMI medium alone. The cells were pelleted by centrifugation and the supernatant was analyzed for calprotectin by ELISA as described above.

### Reactive Oxygen Species (ROS)

Neutrophils, at a concentration of 3 x 10^5^ cells/well, were incubated with the selective FPR1 inhibitor, cyclosporine H (CsH at 10 μΜ), the mitochondrial ROS scavenger, MitoTempo (10 μM), or the NADPH oxidase inhibitor, DPI (25 μM) for 30 min before addition of stimuli, such as R848 (2.5 μg/ml), formyl-methionine-leucyl-phenylalanine (fMLP) (5 μM) or plasma samples from vasculitis patients (*n*=14) and healthy controls (*n*=10) (1:50 dilution) for an additional 60 min. Approximately 80–95% of neutrophils were viable after neutrophil stimulation with plasma samples. To detect ROS generation, DHR 123 (0.5 μM) was added during the last 30 min of incubation, and analysis of ROS was performed by flow cytometry. Data were analyzed by FlowJo (Tree Star Inc, Ashland, OR), and the results were presented as the mean fluorescent intensity (MFI) of ROS.

### Statistics

For samples with a non-Gaussian distribution, non-parametric tests, Mann-Whitney *U* test, Wilcoxon signed-rank test, and Spearman’s correlation were used as applicable. The 95th percentile of healthy controls was used to define the cut-off value for high levels of biomarkers. All analyses were considered statistically significant at *p* < 0.05.

## Results

### Demographic characteristics of the study population

The median age (range) of patients in remission at the time of diagnosis was 47 years of age (10–80) for the GPA cohort, whereas for the MPA cohort it was 59 years of age (17–82). With regards to LVV, the median age (range) of patients in remission was 30 (12–58) and 69 (54–90) years for the TAK and GCA cohorts, respectively. Sixty-four out of 123 (52%) patients with GPA, 31/61 (51%) with MPA, 53/58 (91%) with TAK, and 49/68 (72%) with GCA were female (Tables 1 and 2).

### Neutrophil activation in patients with vasculitis

**Fig. 1:**
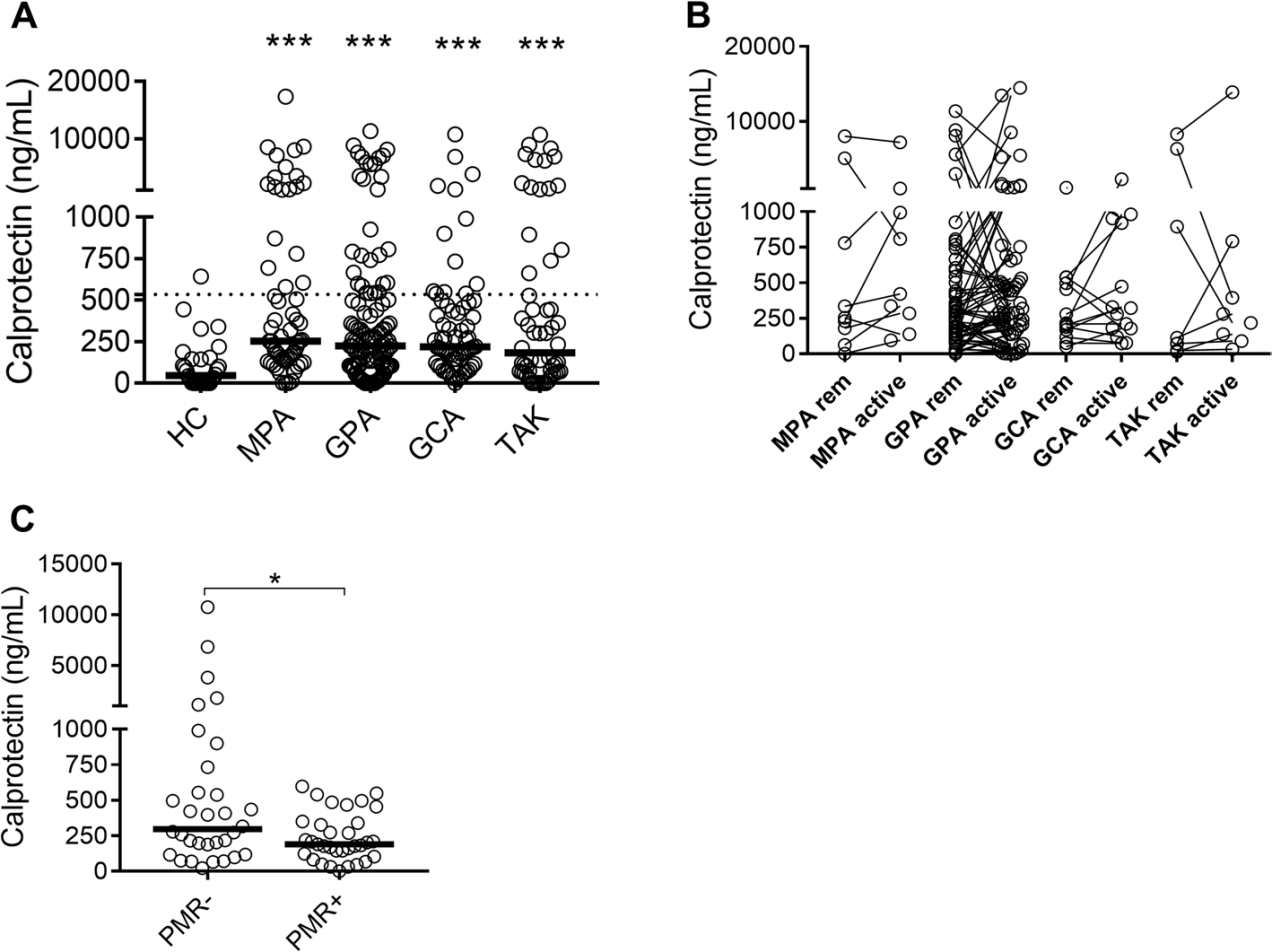
Levels of neutrophil activation marker calprotectin in patients with AAV and LVV. Plasma levels of **A** calprotectin were measured by ELISA in healthy controls (HC), and patients with microscopic polyangiitis (MPA), granulomatosis with polyangiitis (GPA), giant cell arteritis (GCA), and Takayasu’s arteritis (TAK) in remission. Plasma levels of **B** calprotectin were related to disease activity in patients in remission (rem) and matching patients with active disease (active) as assessed by physician global assessment (PGA) in MPA, GPA, GCA, and TAK. Comparison of plasma levels of **C** calprotectin, among patients with GCA in the presence or absence of overlapping diagnosis of polymyalgia rheumatica (PMR): (PMR+, and PMR−, respectively). Statistical analyses were done using Mann-Whitney *U* test (**A** ,**C**), and Wilcoxon signed-rank test (**B**) with *p < 0.05, **p < 0.01, and ***p < 0.001. Unless otherwise indicated, all analyses are compared to healthy controls. Each circle represents an individual sample, with the bar representing the median of the group. The dotted line represents the 95th percentile of the HC

As depicted in Fig. 1A, levels of calprotectin were elevated in patients in remission with MPA (*p*<0.0001), GPA (*p*<0.0001), GCA (*p*<0.0001), and TAK (*p*=0.0004), as compared to healthy individuals, with 20–32% of the patients having highly elevated levels of calprotectin, as determined by the 95th percentile of healthy individuals. Comparing the vasculitis groups, no statistically significant difference could be seen with regards to calprotectin levels, suggesting neutrophil activation occurring at similar levels in both small and large-vessel vasculitis. In all, neutrophil activation is uniformly observed in patients with both large and small-vessel vasculitis.

### Neutrophil activation is not associated with disease activity

No differences were seen in *plasma* levels of calprotectin between patients in remission and flare for any of the vasculitis groups investigated (Fig. 1B). Though not related to disease activity, levels of calprotectin correlated with systemic inflammation, including CRP (*r*=0.20, *p*=0.001) and ESR (*r*=0.24, *p*=0.0001).

We also assessed the correlation between serum creatinine (as a marker of kidney involvement) and calprotectin. Interestingly, levels of calprotectin correlated with creatinine in ANCA-associated vasculitis (MPA: *r*=0.35, *p*=0.01; GPA: *r*=0.41, *p*=0.001), but not in large-vessel vasculitis (GCA: *r*=0.01, *p*=0.92; TAK: *r*=−0.12, *p*=0.42).

Patients with overlapping GCA/PMR disease had significantly lower levels of calprotectin (*p*=0.04), as compared to patients with GCA without the diagnosis of PMR (Fig. 1C). In all, neutrophil activation markers were not associated with disease activity, but rather specific clinical features, such as kidney involvement in patients with AAV, and absence of PMR in patients with GCA.

### Levels of circulating N-formyl methionine peptides in patients with vasculitis

**Fig. 2:**
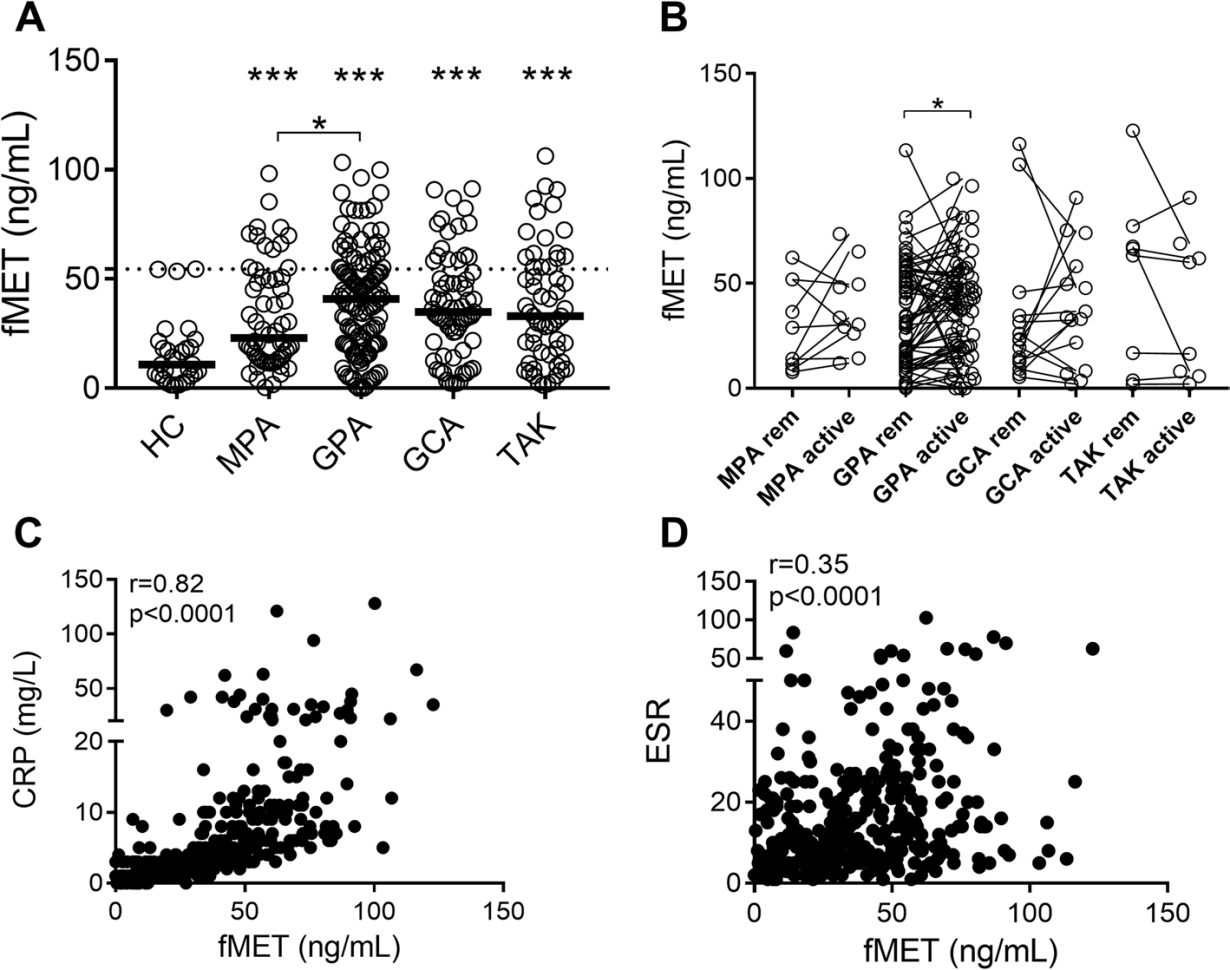
Levels of N formyl methionine peptides (fMET) in patients with AAV and LVV. Levels of **A** fMET were analyzed by ELISA in HC, and patients with MPA, GPA, GCA, and TAK in remission. Plasma levels of fMET (**B**) were related to disease activity in patients in remission (rem) and matching patients with active disease (active) as assessed by PGA in MPA, GPA, GCA, and TAK. Plasma levels of fMET correlated with CRP (**C**) and ESR (**D**). Statistical analyses were done using Mann-Whitney *U* test and Wilcoxon signed-rank test, with *p < 0.05, **p < 0.01, and ***p < 0.001. Each circle represents an individual sample, with the bar representing the median of the group. The dotted line represents the 95th percentile of the HC

As depicted in Fig. 2A, levels of fMET were elevated in all vasculitis groups (MPA, *p*=0.0001; GPA, *p*<0.0001; GCA, *p*<0.0001; and TAK, *p*<0.0001) as compared to healthy individuals, with 22–33% of the patients having highly elevated levels of fMET. Within the different vasculitis groups, fMET levels were higher in GPA as compared to MPA (*p*=0.04).

We also stratified patients based on positivity for either anti-PR3 or anti-MPO antibodies in GPA and MPA. We found that the presence of anti-PR3 antibodies in GPA, or anti-MPO antibodies in MPA, was not associated with elevated levels of fMET (*p*=0.76, and *p*=0.95, respectively) as compared to corresponding patients without these autoantibodies.

Further, in GPA, but not in the other vasculitides, levels of fMET were associated with active disease (*p*=0.02, Fig. 2B). Similarly to calprotectin, levels of fMET were correlated with CRP (*r*=0.82, *p*<0.0001), and ESR (*r*=0.35, *p*<0.0001) (Fig. 2C and D, respectively).

### Neutrophil activation is partially mediated through fMET/FPR1 signaling in AAV and LVV

**Fig. 3:**
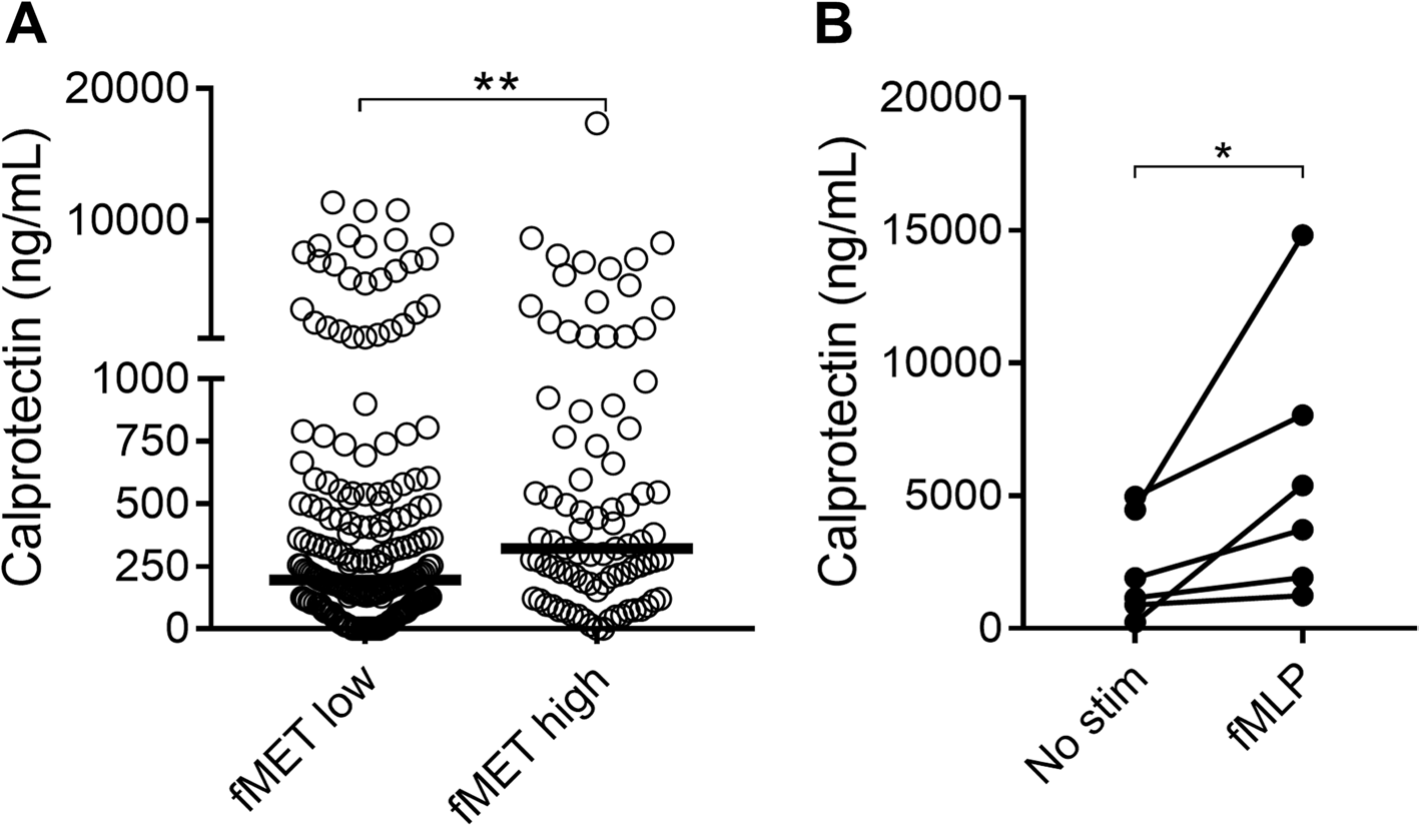
fMET-mediated release of calprotectin. Comparison of plasma levels of **A** calprotectin in patients with either high or low levels of fMET, as determined by the 95th percentile of HC. Neutrophils were activated by fMLP in vitro (**B**) and assessed for calprotectin release by ELISA. Statistical analyses were done using Mann-Whitney *U* test (**A**) and Wilcoxon signed-rank test (**B**), with *p < 0.05 and **p < 0.01. Each circle represents an individual sample, with the bar representing the median of the group

We then investigated whether levels of fMET were associated with markers of neutrophil activation in patients with vasculitis. Levels of fMET correlated with the neutrophil activation marker calprotectin (*r*=0.19, *p*=0.001), and patients with high fMET levels, had markedly elevated levels of calprotectin (*p*=0.002, Fig. 3A). Furthermore, in vitro activation of neutrophils from healthy donors with fMLP, a commonly used N-formyl methionine peptide, resulted in neutrophil activation and subsequent release of calprotectin (Fig. 3B), demonstrating that fMET directly could result in calprotectin release.

**Fig. 4:**
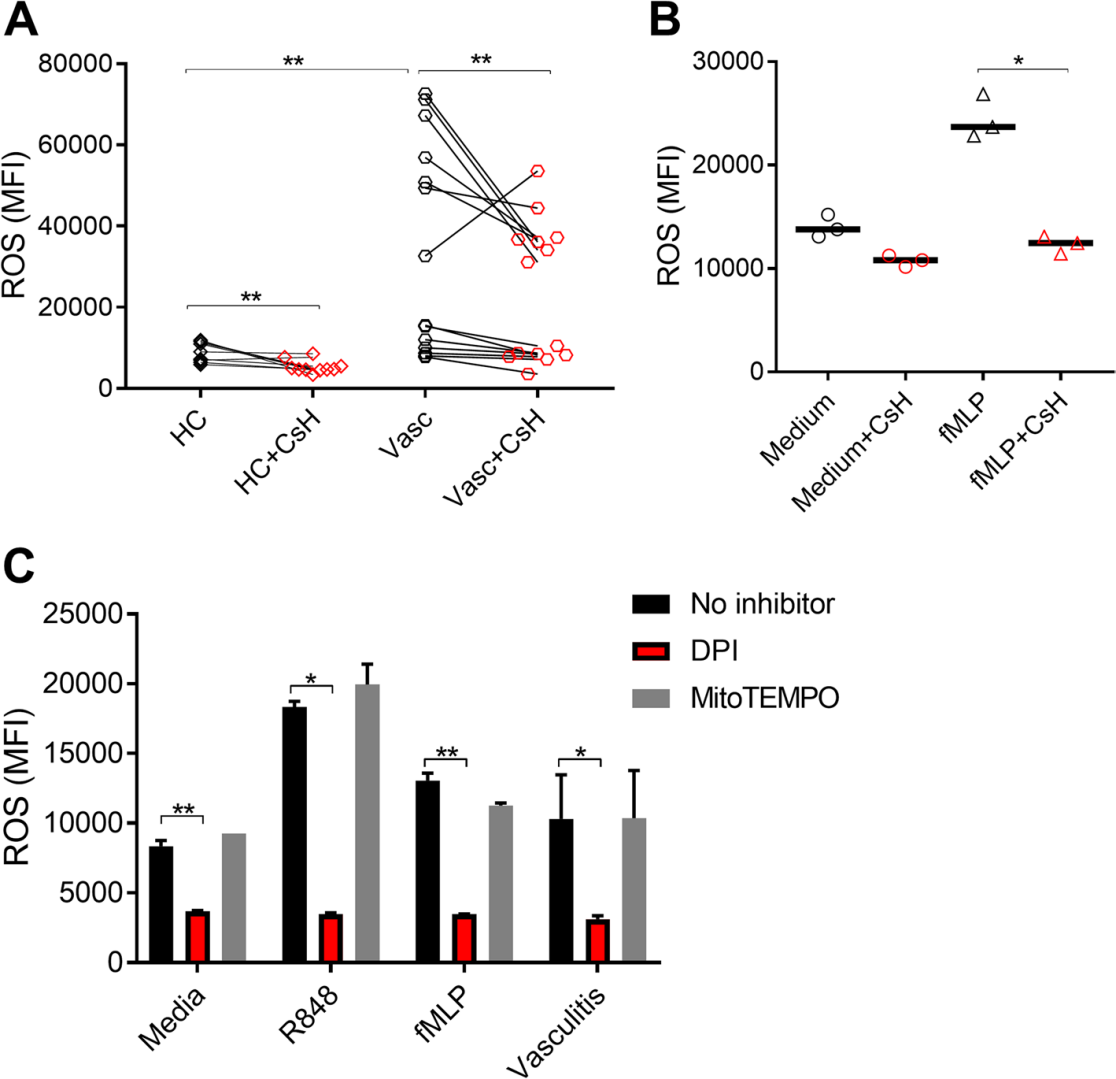
fMET activates neutrophils in an FPR1-dependent manner. Neutrophils from a healthy donor were incubated with **A** plasma from healthy individuals (HC) or patients with vasculitis (Vasc) or **B** medium and fMLP in presence or absence of Cyclosporine H (CsH) and analyzed for ROS induction using flow cytometry. **C** Neutrophils were pre-incubated with inhibitors scavenging mitochondrial ROS (MitoTEMPO) and inhibiting NADPH oxidase (DPI) and analyzed for capacity to induce ROS production upon stimulation with media, TLR8 agonist R848, fMLP, and/or plasma from patients with vasculitis (*n*=10). Statistical analyses were done using Wilcoxon signed-rank test with * *p* < 0.05 and **p < 0.01. Each circle represents an individual sample, with the bar representing the median of the group

To determine whether the circulating level of fMET had the capacity to induce de novo neutrophil activation in patients with vasculitis, neutrophils from healthy individual were incubated with healthy control (HC) and patient plasma and assessed for neutrophil activation as determined by ROS induction. A high proportion of plasma samples (7/14, 50%) from patients with AAV and LVV induced substantial ROS production (Fig. 4A).

Plasma samples potentially contain several neutrophil agonists, including inflammatory cytokines, immune complexes, and not least fMET. To determine whether plasma-mediated neutrophil activation was dependent on fMET in vasculitis, neutrophils were pre-treated with cyclosporine H. Blocking FPR1 with cyclosporine H reduced the capacity for plasma to induce neutrophil activation and ROS production in both HC and plasma from patients with vasculitis (*p*<0.01, Fig. 4A). As a control, CsH was shown to block fMLP-mediated neutrophil activation (*p*<0.05), but not that of medium control (Fig. 4B).

To further discriminate the origin of ROS production, neutrophils were pre-treated with mitoTEMPO and DPI, targeting either mitochondrial ROS or NADPH oxidase, respectively. Blocking NADPH oxidase, using DPI, completely abolished the capacity of TLR8 ligand R848, fMLP, or plasma from patients with vasculitis to induce ROS formation in neutrophils, whereas mitochondrial ROS was dispensable (Fig. 4C). Thus, our findings suggest that plasma from patients with vasculitis induces ROS through an FPR1- and NADPH oxidase-dependent manner.

## Discussion

Mitochondria, due to their prokaryotic origin, contain several immunogenic molecules, including ATP, succinate, cardiolipin, fMET, mitochondrial DNA (mtDNA), and TFAM which, when misplaced from cells such as neutrophils, platelets, or damaged cells into the extracellular space, acquire the immunogenic capacity to drive autoimmune and inflammatory responses [30]. Although the presence of anti-cardiolipin antibodies has been reported in patients with AAV and LVV [31–33], implicating the presence of extracellular mitochondria exposing these immunogenic phospholipids, nothing is known about the presence and the role of other mitochondrial-derived damage-associated molecular patterns (DAMPs), including fMET, in systemic vasculitides.

In the current study, using samples from a well-characterized cohort collected in a protocolized fashion and at several different expert centers, we made the novel observation of fMET seeming to propagate neutrophil activation in both AAV and LVV in a FPR1-dependent manner, the main receptor for fMET [34]. Our findings suggest a central role of mitochondrial components in the pathogenesis of systemic vasculitides through immune activation of neutrophils that produce ROS predominantly from NADPH oxidase, and fMET-mediated signaling as a potential pharmacological target of these rare diseases. Of note, we recently described inhibition of fMET/FPR1 signaling with the selective FPR1 inhibitor cyclosporine H also in patients with RA, suggesting fMET-mediated neutrophil activation possibly being a central process in several autoimmune inflammatory conditions [26].

Consistent with elevated levels of fMET, we made the novel finding that patients with LVV, similarly to AAV, had marked neutrophil activation in peripheral blood, as evident by high levels of calprotectin. Our observation that fMLP can result in release of calprotectin from neutrophils aligns with previous studies that suggested a G protein-coupled pathway as a proposed mechanism [35]. The lack of association with disease activity is in contrast to some prior studies that showed a significant association between serum levels of calprotectin and disease activity in patients with AAV [36] and TAK [37]. However, in another study, serum calprotectin levels did not correlate with disease activity in TAK and GCA and failed to outperform traditional biomarkers such as ESR and CRP in reflecting disease activity in GCA [38].

It must be noted though that previous studies assessed levels of calprotectin in serum [36, 39], and not in plasma as done in the current study, which is an important distinction. We [5, 40], and others [41], have demonstrated that the process of coagulation induces the release of calprotectin. Thus, serum levels do not represent physiological levels of calprotectin, but artificial levels induced upon coagulation. Even so, levels of the neutrophil activation marker calprotectin correlated, though weakly, with markers of systemic inflammation, CRP and ESR, in contrast to prior work on serum levels of calprotectin in GCA [42] and AAV [43], further highlighting the necessity of assessing coagulation-sensitive biomarkers in plasma for consistent, and physiologically relevant, interpretations.

Although levels of neutrophil biomarkers could not distinguish the active disease from inactive disease, calprotectin may be useful as an organ-specific disease damage biomarker in patients with AAV, as it correlated with worsening kidney function in our study. Indeed, in another study, serum calprotectin was suggested as a potential renal prognosis biomarker in AAV [43]. Similar findings have also been seen in lupus nephritis [6, 44]. We also demonstrated that levels of fMET were associated with active disease in GPA. The elevated levels of fMET could be related to dysregulation of cell death pathways, as previous studies have shown that neutrophils derived from patients with GPA have dysregulation in proteinase 3-associated proteins that are associated with apoptosis such as annexin-A1 [45].

Importantly, levels of fMET, and calprotectin, were elevated in some patients with AAV and LVV in clinical remission, suggesting that these levels may indicate subclinical disease activity and smoldering vessel inflammation in systemic vasculitides. Similarly, previous studies showed elevated serum levels of calprotectin in patients in clinical remission with AAV [36], and RA [5] compared to healthy controls. Notably, calprotectin levels remained elevated in patients with GCA that were in treatment-free remission, qualifying it as a candidate marker for vascular inflammation monitoring [41]. Overall, our observations imply ongoing neutrophil activation and silent tissue inflammation in patients that are considered to have an inactive disease. Further studies are needed to determine underlying factors mediating neutrophil activation in otherwise quiescent disease.

Limitations of our study include the great heterogeneity and variability of our patient population, including the use of a variety of therapeutic agents that may have dampened the inflammatory response. Thus, prospective studies are needed to investigate whether immune-suppressive treatment, similar to what has been reported in lupus nephritis [44], can reduce levels of neutrophil activation markers. Another important consideration is that FPR1 is expressed not only by neutrophils but also by other cells that play a significant role in the pathogenesis of vasculitides such as platelets, monocytes, and macrophages, implying their potential activation by mitochondrial DAMPs in the circulation. The source of fMET could not be established in the current study, with fMET being derived from either mitochondria or bacteria. Indeed, one of the most abundant bacteria in the nasal microbiome of patients with GPA is *Staphylococcus aureus* (*S. aureus*) that could be an instigator of immune responses [46]. Bacterial formylated peptides from *S. aureus* are able to induce potent neutrophil chemotaxis in mice [47]. In another study, formylated peptides that were produced by wild-type strain of *S. aureus* were capable of inducing *S. aureus* septic arthritis [48] and a bacterial plasmid-encoded peptide from *S. aureus* was sufficient of causing MPO-ANCA glomerulonephritis in mice [49], illustrating the potency also of bacterial-derived fMET in causing disease. Further studies are warranted to elucidate the origin of fMET.

## Conclusions

Neutrophil activation is prominent in patients with both AAV and LVV. Neutrophil activating factors such as fMET peptides are markedly increased in plasma from vasculitis and enhance inflammation through FPR1-mediated neutrophil activation. Upon validation with animal studies, fMET/FPR1-mediated signaling may be a novel therapeutic target in systemic vasculitides to limit neutrophil-mediated inflammation and tissue damage.

## Data Availability

All data relevant to this study are included in the article.
